# Radiobiological Modeling Based on ^18^F-Fluorodeoxyglucose Positron Emission Tomography Data for Esophageal Cancer

**DOI:** 10.4172/2155-9619.1000190

**Published:** 2014-09-29

**Authors:** Mariana Guerrero, Shan Tan, Wei Lu

**Affiliations:** 1 Department of Radiation Oncology, University of Maryland School of Medicine, Baltimore, 21201, USA; 2 Department of Intelligent Science and Technology, School of Automation, Huazhong University of Science and Technology, Wuhan, China

**Keywords:** ^18^F-FDG PET/CT, Tumor response, Esophageal cancer, Quantitative imaging, Radiation therapy

## Abstract

**Background:**

We investigated the relationship of standardized uptake values (SUVs) to radiobiological parameters, such a 25 s tumor control probability (TCP), to allow for quantitative prediction of tumor response based on SUVs from ^18^F fluorodeoxyglucose (^18^F-FDG) positron emission tomography (PET) before and after treatment for esophageal cancer.

**Methods:**

We analyzed data from 20 esophageal cancer patients treated with chemoradiotherapy (CRT) followed by surgery. Tumor pathologic response to CRT was assessed in surgical specimens. Patients underwent ^18^F-FDG PET imaging before and after CRT. Rigid image registration was performed between both images. Because TCP in a heterogeneous tumor is a function of average cell survival, we modeled TCP as a function of <SUV_R_>, a possible surrogate for average cell survival (<SUV_R_>=<SUV_after_/SUV_before_>). TCP was represented by a sigmoid function with two parameters: SUV_R50_, the <SUV_R_> at which TCP=0.5, and γ_50_, the slope of the curve at SUV_R50_. The two parameters and their confidence intervals (CIs) were estimated using the maximum-likelihood method. The correlation between SUV before CRT and SUV change <SUV_before_ – SUV_after_> was also studied.

**Results:**

A TCP model as a function of SUV before and after treatment was developed for esophageal cancer patients. The maximum-likelihood estimate of SUV_R50_ was 0.47 (90% CI, 0.30-0.61) and for γ_50_ was 1.62 (90% CI, 0-4.2). High initial SUV and larger metabolic response (larger <SUV_before_ –SUV_after_>) were correlated, and this correlation was stronger among responders.

**Conclusions:**

Our TCP model indicates that <SUV_after_/SUV_before_> is a possible surrogate for cell survival in esophageal cancer patients. Although CIs are large as a result of the small patient sample, parameters for a TCP curve can be derived and an individualized TCP can be calculated for future patients. Initial SUV does not predict response, whereas a correlation is found between surrogates for initial tumor burden and cell kill during therapy.

## Introduction

^18^F-fluorodeoxyglucose (^18^F-FDG) positron emission tomography (PET) is routinely used as a tool to diagnose and evaluate response in many cancer sites. A body of published evidence has documented correlation of standardized uptake value (SUV) with tumor clonogenic cell 60 density (or tumor cellularity) and tumor proliferation (e.g., Zhou et al. [1], Fischer et al. [2]). For more than 20 years, reports have specifically documented correlations between ^18^F-FDG uptake changes and response to therapy (e.g., Wahl et al. [3]). In esophageal cancer and gastroesophageal junction tumors, Omloo et al. [4] and Wu et al. [5] found mixed results in terms of SUV correlation with survival and/or pathological response (both for pretreatment SUV and changes in SUV before and after chemoradiation). Even when a correlation between SUV and response is established, in most cases no known quantitative relationship between SUVs and tumor characteristics and response patterns can be identified. ^18^F-FDG PET imaging, therefore, is typically used in a qualitative or semiquantitative manner. The purpose of this work is to investigate the quantitative relationship between mean patient SUVs and radiobiological parameters (such as cell survival and tumor control probability [TCP]) to facilitate quantitative prediction of tumor response based on SUVs from ^18^F-FDG PET before and after treatment. One example in which quantitative knowledge of tumor control probability is essential in determining which patients are suitable candidates for surgery after neoadjuvant chemoradiotherapy (CRT) is that of esophageal cancer. Tan et al. [6] showed that the use of features from the spatial distribution of SUVs gives a more accurate prediction of esophageal cancer patients’ pathological response to CRT than the use of a single SUV value, such as the maximum SUV (SUVmax) within the tumor. That study was based on a group of 20 esophageal cancer patients with pretreatment and posttreatment ^18^F-FDG PET-CT images that were registered using rigid registration, allowing a voxel-to-voxel investigation of changes in SUVs before and after treatment [6]. In this work, we investigate the same cohort of 20 esophageal cancer patients evaluated in our previous study and use the mean values of the distribution of SUV in each patient to obtain a tumor control probability curve (based on pathological response) as a function of mean ratios of SUV before and after CRT.

## Materials and Methods

### Patient cohort and imaging techniques

This Institutional Review Board–approved study was based on a cohort of 20 esophageal cancer patients treated at our institution with trimodality therapy (CRT followed by surgery) from 2006 to 2009. All patients underwent both pre-CRT and post-CRT PET/CT imaging. PET images were attenuation corrected, with a resolution of 4.0×4.0×4.0 mm^3^, and CT images had a resolution of 0.98×0.98×4.0 mm^3^. Rigid image registration (VersorRigid^3^DTransform in the Insight Segmentation and Registration Toolkit 4.6.0; National Library of Medicine, Bethesda, MD) was used to register post-CRT CTs to pre-CRT CTs (details of patient characteristics, imaging protocols, and registration technique can be found in Tan et al. [6]). All patients were treated with external-beam radiotherapy (50.4 Gy; 1.8 Gy/day, 5 days/week) with concurrent chemotherapy consisting of cisplatin and 5-fluorouracil. The tumor volume in the pre-CRT PET image was defined as the region with SUV >2.5 (an SUV of 2.5 has been widely used as an uptake threshold for ^18^F FDG differentiation of benign from malignant lesions in various cancers [7,8]). Surgical resection was performed in all patients 1–7 weeks following the post-CRT PET/CT, and resected specimens was submitted to a pathologist for evaluation. The specimen was semiquantitatively categorized into one of three groups: pathologic complete response (pCR), microscopic residual disease (mRD), or gross residual disease (gRD), according to the amount of residual viable carcinoma observed in relation to areas of fibrosis [8]. In this study, patients with pCR or mRD were considered to be “responders,” because these have been shown to be associated with similar survival rates [9,10]. Patients with gRD were considered to be “nonresponders.”

### Radiobiological modeling

We first considered a Poissonian TCP model: TCP=exp(−NoS), where No is the total initial number of clonogenic cells and S is the survival fraction after CRT treatment. As other authors have proposed [11,12], we want to develop a TCP model based on SUV signal from FDG PET images. For simplicity we did not explicitly include a repopulation term in TCP; however, the survival fraction S can be thought of as an “effective survival” that implicitly accounts for the repopulation effect. For a tumor with inhomogeneous response that has Nc compartments 120 with different cell survival after CRT and S(k) in each compartment k, TCP can be expressed as:

(1) $$\mathrm{TCP}=\prod_{K=1}^{N_{c}}e^{-N_{o}(K)S(K)}=e^{-\sum_{k=1}^{N_{c}}N_{o}(K)S(K)},$$

where N_o_(k) is the initial number of clonogenic cells in compartment k with volume vk and can be written as N_o_(k)=N_o_f(S(k)) with f(S(k)) representing the fraction of cells with survival fraction S(k) (equivalently the fraction of clonogenic cells in compartment k). Substitution of N_o_(k)=Nof(S(k)) in Eq. (1) yields:

(2) $$\mathrm{TCP}=e^{-N_{o}\sum_{k=1}^{N_{c}}f\left(S(K)\right)S(K)}\underset{N_{c\to \infty ,V_{k}\to 0}}{\to }e^{-N_{o}\int f\left(S\right)\mathrm{SdS}}=e^{-N_{o}\langle S\rangle }$$

where <S> is the average survival over f(S), the distribution of survival fractions across the tumor. Equation 2 shows that if a Poissonian TCP is assumed, then TCP depends on the average survival in the tumor for any arbitrary inhomogeneous distribution of survival fraction f(S). The problem of inhomogeneous response to radiation has been studied in the context of modeling hypoxic regions in tumors (e.g., Lind and Brahme [13]). Some authors have assumed a distribution of radiosensitivities SF2 (survival fraction at 2 Gy) with two compartments, one radiosensitive and one radioresistant [13]. Equation 2 is valid for any arbitrary distribution of survival fractions S, and no specific relation with SF2 is assumed, because S represents effective survival after treatment with both radiation and chemotherapy. In order to use Equation 2 for TCP it is necessary to find a surrogate for average survival fraction using SUV PET values from before and after treatment. If we assume that the SUV value in a voxel j of the ^18^F-FDG 140 PET images correlates with the number of clonogenic cells in that voxel, it follows that the ratio of SUV values after and before CRT (i.e., SUV_R_(j)=SUV(j)_after_/SUV(j)_before)_ can potentially be a surrogate for the survival fraction in that voxel [14]. Therefore, the average of SUVR(j) over all the voxels in the tumor delineated on pre-CRT PET can potentially be a surrogate for the average survival fraction in the tumor. We calculated the average <SUV_R_>=<SUV_after_/SUV_before_> for each patient. We also calculated other possible surrogates, such as <SUV_after_>/<SUV_before_> and <SUV_before_ − SUV_after_>. The mean values were calculated in the tumor region (defined in the PET images before therapy with SUV >2.5). For a possible surrogate of the average survival fraction to be useful, its values for responders must be significantly different from the values for nonresponders. This was evaluated by comparing the average values of each possible surrogate for responders and nonresponders with a t test at a significance level ≤ 0.05.

### Maximum-likelihood estimate of TCP model parameters

**Figure 1:**
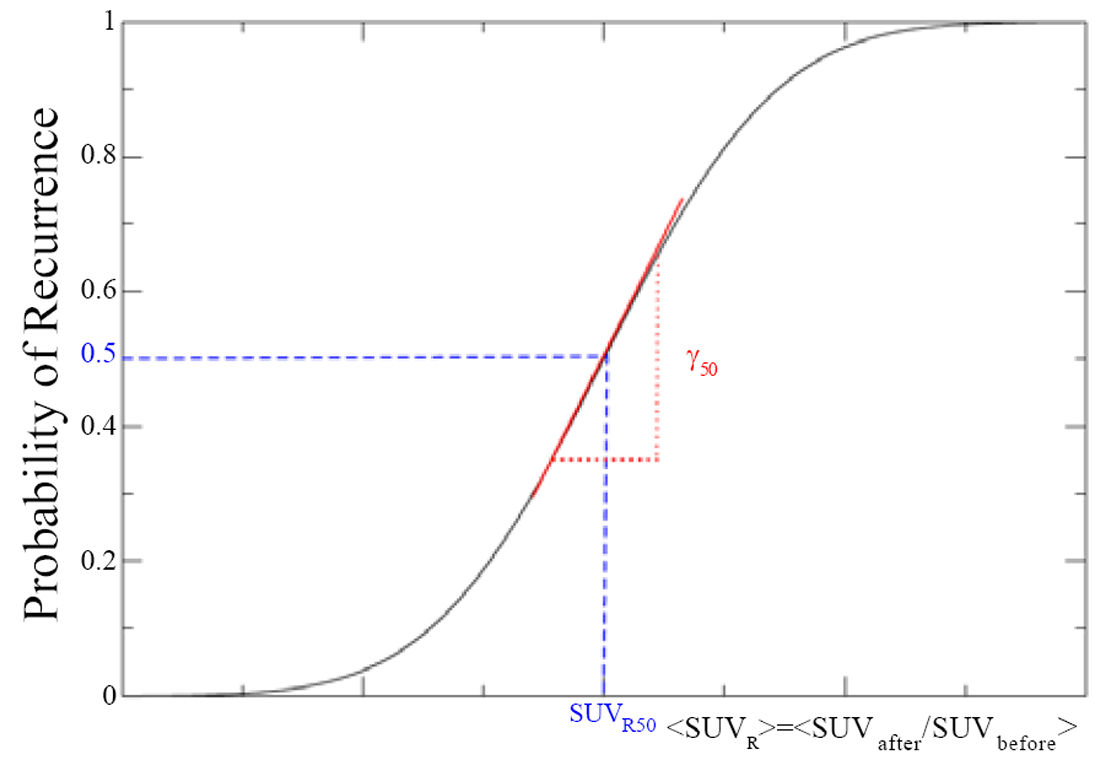
The sigmoid function representing the probability of recurrence as a function of <SUV_R_> is characterized by two parameters: SUV_R50_ and γ_50_.

Although <SUVR> as defined above is a reasonable surrogate for average survival fraction, the explicit functional dependence of <SUVR> with survival fraction is not known, so we cannot directly use Eq. (2) to relate TCP and <SUVR>. We propose the use of a sigmoid function to relate TCP with <SUV_R_>, as a reasonable starting point. The sigmoid function is widely used to model TCP and normal tissue complication probability (NTCP) as a function of dose and was used previously to model tumor control probability based on PET images [11]. Figure 1 is a graphical representation of a typical sigmoid function.

It is constrained to the interval (0–1) and typically defined by two parameters: D_50_, the dose at which TCP is 0.5, and γ_50_, the normalized slope of the (sigmoid) curve at D50. Similarly, we define SUV_R50_ as the value of <SUV_R_> at which TCP is 0.5 and γ_50_ as the slope of the curve at SUVR50. For convenience we define the tumor recurrence probability (TRP) as 1–TCP, which is characterized by the same parameters (the slope simply changes sign) as TCP. For the functional representation of a sigmoid-shaped. TRP we use the error function:

(3) $$\emptyset_{\mathrm{SU}V_{R_{50,}\gamma_{50}}}(\mathrm{SU}V_{R})=0.5\;\left[1+\mathrm{erf}((\mathrm{SU}V_{R}-\left(\mathrm{SU}V_{R50}\right)\gamma_{50}\sqrt{\pi }\right]$$

where the error function is the standard definition:

(4) $$\mathrm{erf}\;\left(\times \right)=\frac{2}{\sqrt{\pi }}\int_{0}^{x}e^{-t^{2}}\mathrm{dt}$$

To determine the parameters SUVR50 and γ50 that best fit our data, we use the maximum-likelihood estimate method, which is often used to determine TCP and NTCP parameters based on clinical data (e.g., as in Dawson et al. [15]). The maximum-likelihood estimate method can be summarized as follows. Each patient in the group has a specific value of <SUVR> calculated from his or her PET images. For given values of SUV_R50_ and γ_50_, the probability of tumor recurrence for each patient I is expressed as:

(5) $$P_{i}=\emptyset_{\mathrm{SU}V_{R_{50},\gamma_{50}}}\left(\mathrm{SU}V_{\mathrm{Ri}}\right)$$

The log-likelihood (LL) can be calculated as:

(6) $$\mathrm{LL}\left(\mathrm{SU}V_{R50},\gamma_{50}\right)=\sum_{i}\log{\left(p_{i}\right)}^{R_{i}}+\log{\left(1-p_{i}\right)}^{\left(1-R_{i}\right)}$$

where R_i_=1 if the patient is a nonresponder and R_i_=0 if he or she is a responder [15]. The most likely values for SUVR_50_ and γ_50_ are obtained by maximizing LL(SUV_R50_, γ_50_). The confidence intervals (CI) for SUVR_50_ and γ_50_ can be estimated using standard statistical methods assuming a Gaussiandistribution with two degrees of freedom (e.g., as in Beringer et al. [16]).

## Results

**Figure 2:**
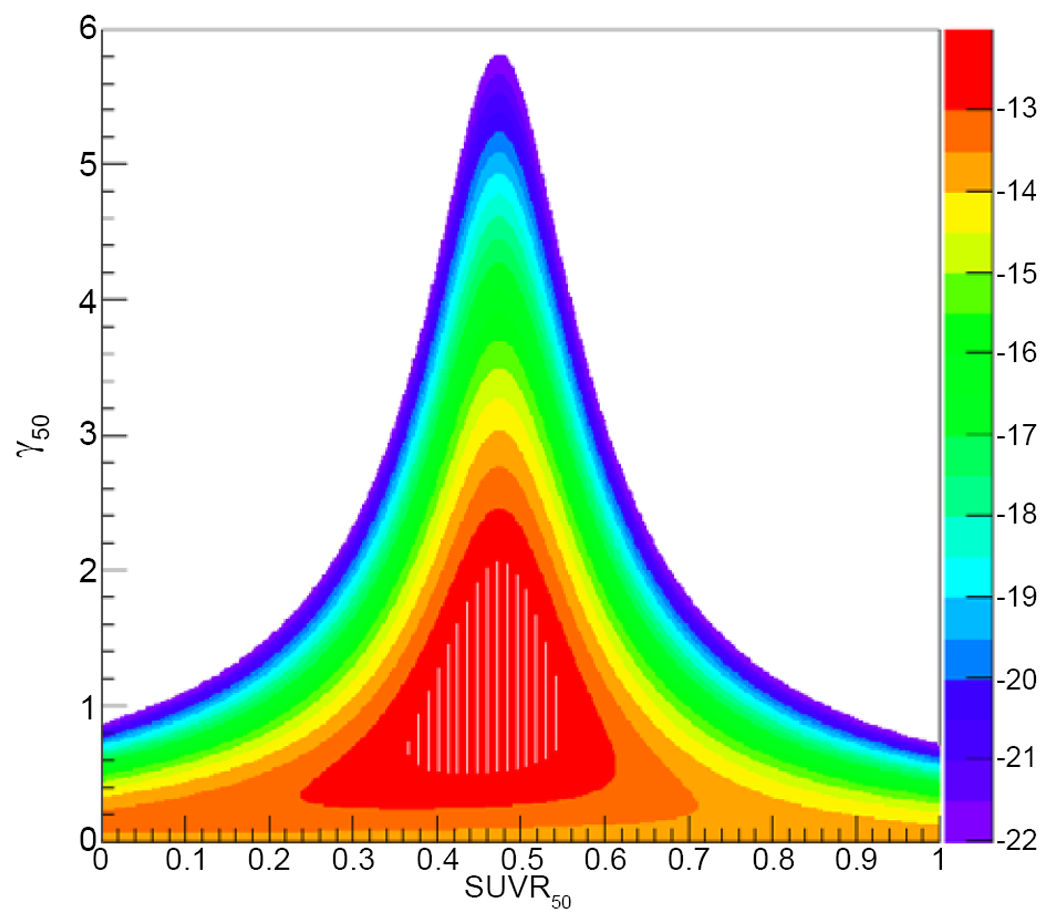
Two-dimensional plot of the log-likelihood as a function of SUV_R50_ and γ_50_. The most likely parameters are defined by the maximum log-likelihood.

Table 1 we present the average values of each of the possible survival fraction surrogates for responders and nonresponders, as well as standard deviations and their P values. Table 1 shows that <SUV_after_/SUV_before_>, <SUV_after_>/<SUV_before_> and <SUV_before_−SUV_after_> each have significantly different values for responders and nonresponders (P<0.05). The fact that <SUVafter/SUVbefore>, <SUV_after_>/<SUV_before_> and <SUV_after_−SUVbefore> are significantly different for responders and nonresponders validates these quantities as good candidates for surrogates of the effective survival fraction. Here we present our results by calculating TRP as a function of <SUV_after_/SUV_before_> to illustrate the method. Equivalent results can be obtained using <SUV_after_>/<SUV_before_> or <SUV_after_−SUV_before_>. Figure 2 is a two dimensional plot in which the x and y axes represent SUV_R50_ and _γ50_, respectively, and the color scale shows the LL calculated using Equation 6.

**Figure 3:**
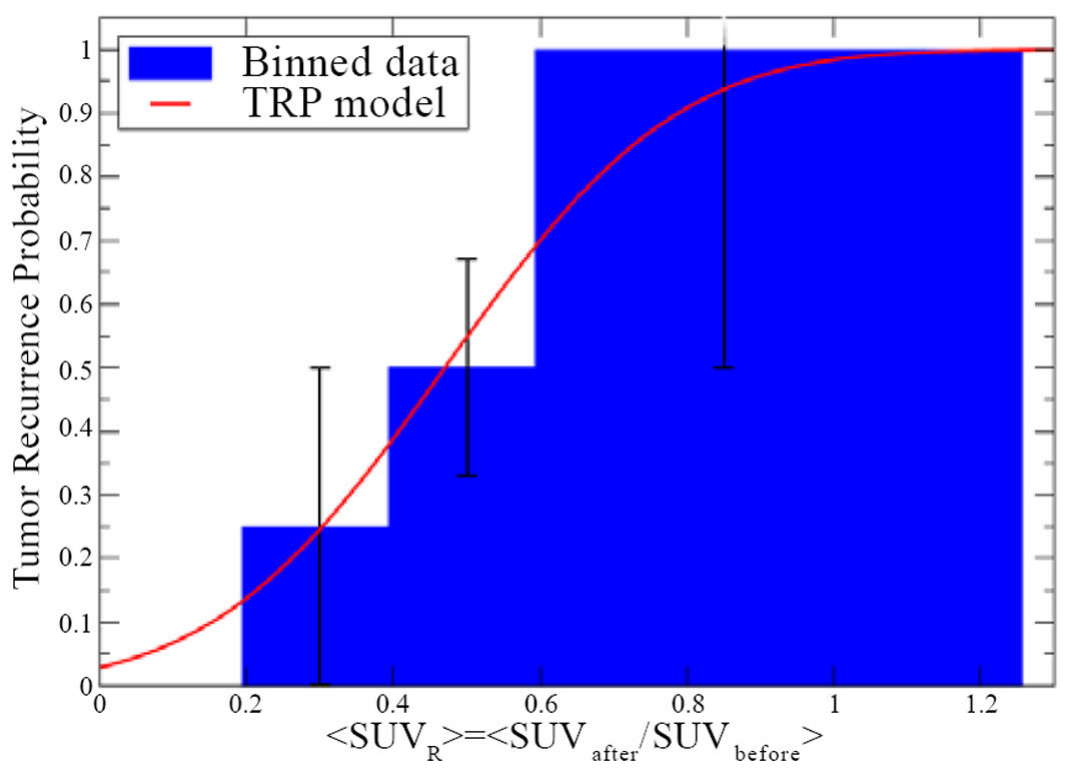
Tumor recurrence probability model compared to data represented in a histogram with 3 bins. The model reasonably represents the data despite large error bars resulting from the small patient sample.

The maximum LL value is at SUV_R50_=0.47 (90% CI, 0.3-0.6) and γ50=1.61 (90% 210 CI, 0-4.2). To compare the model with our patient data, we divided our patients’ <SUV_R_> results into three bins (0.2-0.4; 0.4-0.6; and >0.6). Based on the numbers of responders and nonresponders in each group we plotted the histogram with the TRP and compared it with the model prediction (Figure 3). Although the error bars are large (as well as the confidence interval for the parameters), Figure 3 shows that the model describes the data reasonably well and illustrates the way in which a TRP and, equivalently, a TCP as a function of SUV can be derived from clinical data.

**Figure 4:**
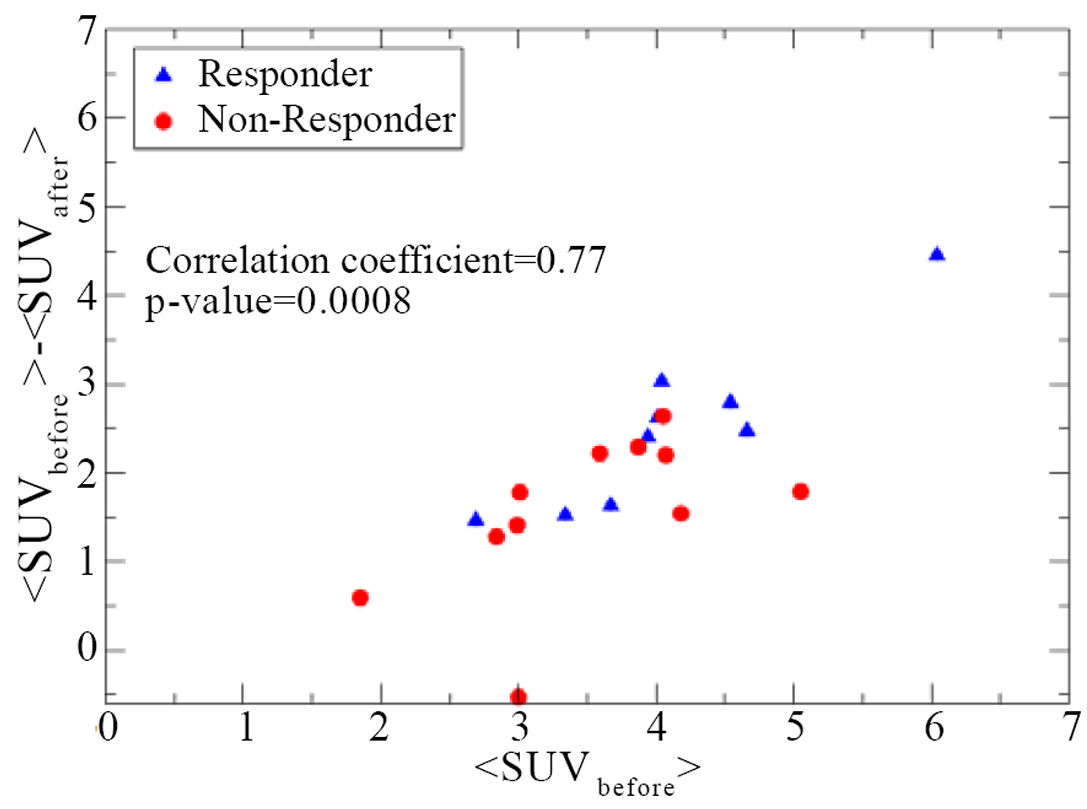
Scatter plot of the difference <SUV_before_> − <SUV_after_> as a function of <SUV_before_>. A significant correlation is seen among those quantities.

Table 1 also shows that <SUV_before_> tends to be higher for responders than for non responders. This trend, although not significant, seems to contradict the assumption that SUV is correlated with tumor burden, because we expect nonresponders to have higher tumor burdens than responders. This trend has been observed before in studies of initial SUV_max_ for lung cancer [17] as well as in esophageal cancer [18]. To address this puzzling issue, we studied the correlation of <SUV_before_> with <SUV_after_/SUV_before_> and <SUV_before_−SUV_after_> to determine whether the response surrogates are correlated with the initial SUV values. Figure 4 shows a significant correlation between <SUV_before_> −<SUV_after_> and <SUV_before_> (Pearson correlation coefficient=0.77; P=0.0008).

This correlation was even stronger among responders (correlation coefficient=0.92; P=0.005). A correlation trend between <SUV_R_> and <SUV_before_> was also found but did not reach statistical significance.

## Discussion

This work presents a proof of principle for a method to quantitatively relate the ratio of mean SUV after and before treatment to the probability of tumor recurrence in patients with esophageal. To the best of our knowledge, this is the first study where a quantitative relationship between average SUV before and after treatment and tumor control probability has been developed.

Although our confidence intervals are large as a result 245 of the small sample size, our example shows that a patient-specific TRP curve (as depicted in Figure 3) can be derived with this method. This TRP curve could potentially be used to estimate the probability of disease recurrence after CRT given the value of <SUV_R_> for a given patient, which could help in a personalized medicine approach to determine the need for subsequent surgery. Instead of looking for an arbitrary cut-off in SUVs and determining the sensitivity and specificity of a positive or negative test, our method uses a continuous TRP curve and offers the advantage of easily identifying patients for whom PET imaging response results should be labeled as inconclusive. In our example, patients with <SUV_R_> between 0.3 and 0.6 (CI for SUV_R50_) have a 50/50 chance of recurrence. In that case, <SUV_R_> should not be used as a determining factor for sending the patient to surgery. A limitation of our approach is that the patient number was small and a larger patient population may be needed to obtain TCP values with reasonably small CIs to clinically validate the model parameters. We used a sigmoid function to represent the TRP because it is a common choice for TCP versus dose and it is restricted to values from 0 to 1. The sigmoid function also has the property that for a steep slope it reproduces a step function, which is commonly used to report ^18^F-FDG PET imaging results.

The derivation of the radiobiological model rests in part on the assumption that SUVs are correlated with tumor burden. This assumption has been a topic of investigation in a number of studies for esophageal cancer that have shown mixed results. In an extensive review of ^18^F-FDG-PET parameters as prognostic factors in esophageal cancer, Omloo et al. [4] found that 12 of 15 studies showed that although pretreatment ^18^F-FDG uptake is a predictor for survival in univariate analysis, only 2 studies showed such uptake to be a predictor of survival in multivariate analysis. In our study, we did not find a statistically significant correlation between initial SUV and pathological response; in fact, we identified a small trend showing higher initial SUV_s_ for responders. Rizk et al. [18] found that pretreatment SUV was a significant predictor of survival for patients managed with surgery only (low SUV, greater survival). However, in a subsequent report, Rizk et al. [19] found that pretreatment SUV_s_ did not predict survival for patients treated with chemoradiation, in part because of the fact that patients with higher pretreatment SUVs responded better to therapy than those with lower SUVs. In a study of 103 patients Brown et al. [20] also found that high initial ^18^F-FDG SUV on PET in esophageal cancer patients was a predictor of survival only for those treated with surgery; in patients treated with neoadjuvant therapy this difference disappeared, and a trend toward better survival was seen in patients with higher initial SUV. These results are consistent with our findings, in which we identified a trend toward higher initial SUV in the CR group and a correlation between better response and higher initial mean SUV, in agreement with the results of Rizk et al. [18]. Whether pretreatment SUV is associated with better outcomes, tumor cell density, or tumor proliferation is not a crucial assumption for our current work. The key assumption in our modeling is that the ratio of <SUV_after_>/<SUV_before_> or the difference <SUV_before_>−<SUV_after_> are surrogates of mean effective cell survival in the tumor.

The correlation of changes in SUV uptake after chemoradiation has been studied by several groups with mixed results: 4 of 10 studies in the review by Omloo et al. [4] found such correlation. Most of these studies, however, relied on SUV_max_ rather than the mean SUV, and some focused on survival as an endpoint rather than pathological response. Tan et al. [6] showed that using the average values (and other features of the distribution) can improve the predictive accuracy of ^18^F-FDG PET in esophageal cancer. Our study showed a significant correlation of pathological response with changes in average SUV. Other groups have also found that considering the spatial extent properties of SUVs can increase predictive accuracy [21,22]. We used <SUV_R_> as our surrogate for cell survival, but other possible surrogates quantifying change in SUV could be used, for example <SUV_before_>−<SUV_after_> or the ratio of the means of <SUV_after_>/<SUV_before_>, because both parameters are significantly different for responders than nonresponders. Moreover, this method could potentially be applied in other imaging modalities when a parameter is significantly different for responders and nonresponders. We believe that the development and validation of quantitative models of TCP as a function of molecular imaging markers will advance the understanding of the radiobiology of those markers.

The correlation of <SUV_before_> − <SUV_after_> with <SUV_before_> shown in Figure 4 underscores the complexity interpretation of SUVs. If SUV is representative of tumor burden, responders would be expected to have smaller <SUV_before_>; the result (Table 1) shows a trend that is opposite to this reasoning. Although changes in SUV before and after treatment are typically used to characterize response (e.g., as in Aerts et al. [23]), it is believed that tumor regions with higher initial SUVs are at higher risk of recurrence (the basis of dose painting strategies). However, the fact that in our group of esophageal cancer patients subjects with higher <SUV_before_> tended to have larger decreases in SUV as represented by <SUV_before_> − <SUV_after_> (a stronger response) shows that simple interpretations may not work because of the complex correlations among radiobiological parameters. As discussed above, other groups have also found correlations between initial SUV and response to chemoradiation [19-21] but those studies focused on either pathological response or patient survival as endpoints. To the best of our knowledge, this is the first study to investigate and find a correlation between initial average SUV and change in average SUV before and after treatment for esophageal cancer patients. Our result helps explain the findings from previous investigators as discussed above [18-21] and make the case for the need of systematic studies of these correlations to help understand and improve the interpretation of ^18^F-FDG PET images as 325 well as other molecular imaging markers.

## Conclusions

The TCP model was characterized using SUV in tumor before and after therapy. According to the TCP model, <SUV_after_/SUV_befor_e> is a possible surrogate for cell survival in esophageal cancer patients. Despite the fact that CIs are large because of the small patient sample, parameters for a TCP curve can be derived and an individualized TCP can be calculated for future patients. Initial SUV did not predict for response, and a correlation was found between surrogates for tumor burden and cell kill.

## Figures and Tables

**Table 1:** Mean values ± standard deviations of possible surrogates for cell survival for responders and non responders with the corresponding P values.

	Responders	Nonresponders	*P* value
**<SUV_after_/SUV_before_>**	0.45 ± 0.11	0.60 ± 0.24	0.04
**<SUV_after_>/<SUV_before_>**	0.41 ± 0.11	0.57 ± 0.23	0.03
**<SUV_after_-SUV_before_>**	2.49 ± 0.93	1.57 ± 0.90	0.02
**<SUV_befor_e>**	4.1 ± 0.94	3.50 ± 0.87	0.08
**<SUV_afte_r>**	1.62 ± 0.38	1.97 ± 0.85	0.13
